# Rapid diagnostic testing as an indicator of malaria prevalence in Rorya District, Tanzania

**Published:** 2021-07-01

**Authors:** Will R. Geisen, Cheryl Bartone, Deborah Gerdes, Christopher Lewis

**Affiliations:** 1 The Christ Hospital, Department of Internal Medicine, Cincinnati, OH, USA; 2 The Christ Hospital, Department of Heart Failure, Cincinnati, OH, USA; 3 University of Cincinnati, Department of Family Medicine, Cincinnati, OH, USA

## Abstract

**Background:**

Rapid Diagnostic Testing (RDT), a point-of-care, qualitative test for *Plasmodium* antigen, has been a catalyst in the diagnosis of patients in malaria-endemic regions. While blood-smear microscopy remains the gold standard, RDT allows for swift diagnosis in resource-poor settings. Our study sought to utilize RDT to quantify local malaria prevalence in the Rorya district of Tanzania.

**Materials and Methods:**

Two field clinics were established and 1,032 patients were screened. Those that described malaria symptoms were tested via RDT. The percentage of positive tests was compared to national data from the World Health Organization’s 2019 World Malaria report and the President’s Malaria Initiative Report for Tanzania. Intake data (sex, age, heart rate (HR), and temperature) were compared between the malaria-positive and malaria-negative groups.

**Results:**

772 patients received RDT of whom 487 tested positive. There was a statistically significant difference in the percentage of positive patients between the two sites (52.0% vs 38.2%). Sixty percent of malaria-positives were female and the median age of this group was 10 yrs (range 5-15 yrs). Intake data showed a notable difference in median heart rates between malaria-positive and malaria-negative persons, 84.0 (72-100) and 72.0 (74-84) beats per minute (bpm), respectively.

**Conclusions:**

The prevalence of malaria in Rorya was significantly higher than the reported Tanzanian average. Additionally, children were at a statistically higher risk of contracting malaria. Our data indicates that RDT offers enhanced insight into the local malarial burden that may be valuable to (governmental) health providers for the disbursement of resources in malaria-endemic regions.

## Background

In 2018, the World Malaria Report estimated 228 million cases of malaria with approximately 405,000 deaths worldwide. Children under five yrs of age were the most vulnerable age group, accounting for 67% of the estimated malaria deaths in 2018. Sub-Saharan Africa, where *P. falciparum* is endemic, has a disproportionally high disease burden with 93% of all malaria cases and 94% of all deaths worldwide. Tanzania, with a population of 56 million, has high malarial morbidity and mortality. Approximately 7,7 million Tanzanians develop malaria annually and, with a prevalence of 114 cases per 1,000 persons (11.4%), Tanzania ranks 28^th^ highest in national malaria prevalence. The report estimated that Tanzania accounted for approximately 2% of malaria cases and 5% of malaria deaths worldwide [1].

Twenty-six percent of all medical visits in Tanzania in 2018 were due to malaria, resulting in approximately 350,000 hospital admissions and 4,000 deaths [2]. These numbers have significantly improved from 2006, when the country experienced 800,000 hospitalizations and 20,000 deaths attributed to malaria [1]. Advances in the prevention of malaria (insecticide treated bednets (ITNs), prophylactic treatment of pregnant women (IPTp)), improvements in detection of *P. falciparum* (rapid diagnostic testing or RDT), and earlier treatment with anti-malarial medication (artemisinin-based combination therapies or ACTs) have allowed Tanzania to experience the aforementioned decrease in disease burden. Both domestic and international campaigns have played key roles in patient education, empowering healthcare providers, distributing supplies, and allocating treatment to vulnerable populations. Perhaps the biggest catalyst in the battle against malaria has been the advent of the RDT. RDT is a point-of-care immunochromatographic lateral flow device that provides a qualitative diagnosis of malaria. The device detects plasmodiumspecific antigens in the patient’s blood, such as histidine rich protein-2 (HRP2), and compares the sample (S) to a negative control (C) [3]. The test results within 20 min and is interpreted similar to an over the counter pregnancy test, with the appearance of ‘C’ and ‘S’ bars indicating the presence of *Plasmodium* species and the appearance of a single ‘C’ bar indicating the absence of plasmodial antigen. Thick smear microscopy remains the gold standard for malaria diagnosis, but RDT offers quick and reliable data in resource poor settings in which electricity, let alone microscopy and trained pathologists, is scarce [4]. Quality control data of RDT has demonstrated that, compared to thick smear and polymerase chain reaction (PCR), RDT is 98% and 97% sensitive, respectively [3]. Therefore, RDT offers prompt, intuitive, and non-inferior diagnostic data in settings in which definitive diagnosis was previously difficult.

Many of Tanzania’s endemic regions are considered resource poor. While larger communities such as Dar es Salaam, Mwanza, and Arusha have adequate access to medical care akin to many Western nations, smaller rural communities remain under-served. These communities experience higher all-cause under-five mortality and malaria is believed to be a significant driver in these preventable deaths. The Rorya district in northwest Tanzania, situated along the shores of Lake Victoria and bordering Kenya, is one of these vulnerable regions, based on risk stratification and high prevalence of malaria [5].

**Figure 1:**
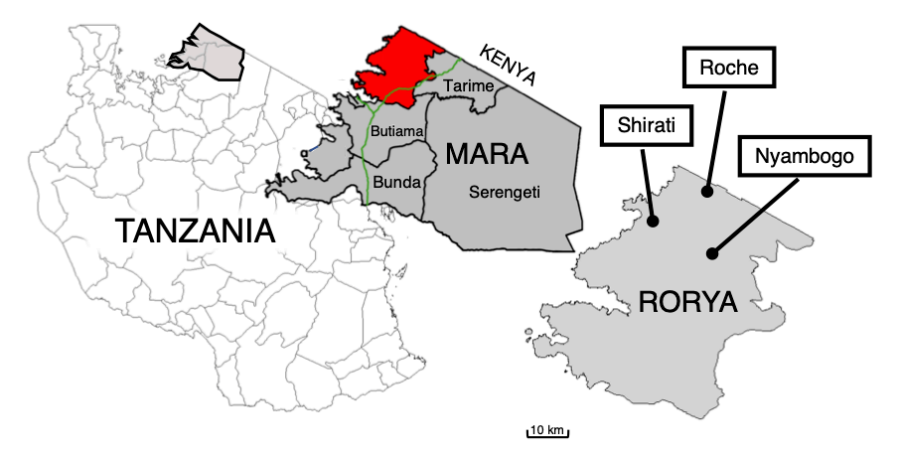
Tanzania (left), the Mara region (middle) showing Rorya District in red and the location of the Roche and Nyambogo health centers, and Shirati (study headquarters) in Rorya District (right).

In March 2020, health providers associated with the Village Life Outreach Project (VLOP) established two medical clinics in the Rorya district, Mara region, of northwestern Tanzania. The two sites, Nyambogo and Roche (Figure 1), worked with community leaders and medical authorities to provide health screening and treatment for over one thousand community members. Malaria testing via RDT and treatment with ACTs accounted for a high proportion of the medical therapy provided at the two sites. Through a uniquely designed, mobile electronical medical record (EMR) system, patients were identified and tracked through their clinic visit (intake, provider encounter, malaria testing, and pharmacy). Subsequently, patient vitals, incidence of malarial symptomatology, the number of patients tested via RDT, the number of positive tests, and the quantity of ACTs distributed to the patients were computed through this EMR system. This study attempted to accurately quantify the prevalence of malaria and the associated disease burden in the Rorya region of Tanzania.

## Materials and Methods

The two clinics were staffed with volunteers including: six medical residents, six pharmacy students, four medical students, six nursing students, eight registered nurses, two pharmacists, and four attending physicians. Clinic workers were distributed evenly between the two clinical sites and switched sites daily to ensure patient/ pathology variability. Additionally, different pairings of workers were formulated each day prior to dispersal. Tri-lingual (English, Swahili, Luo) translators were utilised to obtain biographic information, assist in evaluation of patients, and counsel them regarding pharmacotherapy, referrals, and medical follow up. If patients described a constellation of symptoms consistent with malaria (fever + headache, fever + myalgia, fever + abdominal pain, etc.), the provider would place the malaria testing order in the EMR system. These patients were taken to the RDT station prior to matriculation to the pharmacy. Because there were a limited number of RDT test kits, patients with low probability of malaria skipped RDT and were escorted directly to the pharmacy.

In the RDT station, a small blood sample was collected via finger-prick and was transferred to the RDT device using a curved glass pipet. Buffer solution was added to the sample input. The kit was then allowed to rest undisturbed in a low light setting for 20 min according to manufacturer (Standard Diagnostics, SD Bioline Malaria Ag P.f ®) instructions. The tests were then interpreted and catalogued within the EMR system. When a positive test was identified, the EMR automatically populated a prescription of ACT (Strides Shasun ®, artemether-lumefantrine 20 mg/ 120 mg) for the patient linked with the positive test. The patient would then receive the ACT prescription from the pharmacy after counseling from a licensed pharmacist and tri-lingual translator. The first dose of ACT was taken in-pharmacy under direct supervision prior to cessation of the encounter. The catalogued data was stratified into patients that warranted malaria testing versus those that did not warrant testing. Those patients that warranted testing were further sorted by age, sex, and presenting clinic site. Heart rate (HR) and temperature data were collected during patient check-in and was compared between the malaria-positive and malaria-negative groups. The number of positive tests was quantified and compared to national data from the World Health Organization’s 2019 World Malaria report and the USAID President’s Malaria Initiative Report for Tanzania.

## Results

A total of 1,032 patients were triaged, screened, and treated by the VLOP team in the Rorya district over an 8-day period. Of these, 671 patients reported to the Nyambogo clinic and 361 to the Roche clinic. Of all patients seen, 647 (62.8%) were female and 385 (37.2%) were male. The Nyambogo and Roche clinic sites had a similar percentage of female patients with 62.4% (419 females out of 671 patients) and 63.1% (228 females out of 361 patients), respectively (Table 1).

**Table 1:** Sample data from the 2 clinical sites (Nyambogo and Roche). A statistically significant difference between the sites was noted for median age and number/percentage of malaria-positive patients.

	Nyambogo	Roche	P-value
Total Patients	671	361	
Male	252	133	
Female	419	228	
Median Age (range) yrs	20.0 (9.0-48.0)	17.0 (7.0-38.0)	0.012
Patients Tested	513	259	
% of Patients Tested	76.3	71.9	
Patients w/ + RDT	349	137	<0.001
% of Patients w/ + RDT	52.0	38.2	<0.001

A wide range in patient age was seen, ranging from less than one month old (catalogued 0.1-yr-old) to 92 yrs. The average age of those evaluated was 26.7 yrs. The age distribution at both sites was skewed towards younger persons, so the median range of ages at each site was calculated via the Wilcoxon-Rank sum procedure. The median of age of Nyambogo patients was 20.0 (9.0-48.0) while the median at the Roche site was 17.0 (7.0-38.0) (P=0.012). Nearly 75% of patients (n=772) reported with symptoms consistent with malaria (fever + headache, fever + myalgia, fever + abdominal pain, etc.) that warranted RDT. Within the tested group, 487 persons (63.1%) were positive; hence, 47.2% of the total patient population tested positive for malaria. A significantly (P<0.001) higher percentage of malaria-positive patients was noted in Nyambogo (52.0%; 349/671 persons) versus Roche (38.2%; 137/361 persons; Table 1).

**Figure 2:**
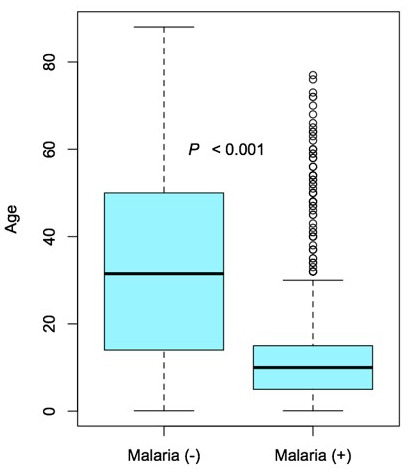
Box and whisker diagramme of the noted significant difference (P<0.001) in median age (bold horizontal line) of the malaria-negative (left) and malaria-positive (right) groups. The interquartile range (box), interquartile range x 1.5 or furthest datum (dotted lines), and any outliers (open circles) are shown.

The gender breakdown of those with active infection was 60.6% female (n=295) and 39.4% male (n=192; P=0,184). The median age of malaria-positive patients was 10.0 yrs old (5.0-15.0) while those that were malaria-negative was 31.5 yrs old (14.0-50.0) (P<0.001) (Table 2, Figure 2).

**Table 2:** A breakdown of the malaria-positive and malaria-negative groupings. A statistically significant difference between the two subsets was observed for median age and heart rate (HR).

	Malaria +	Malaria -	P-value
Total Patients	487	285	
% Female	60.6	68.4	0.184
% Male	39.4	31.6	0.184
Median Age (range) yrs	10.0 (5.0-15.0)	31.5 (14.0-50.0)	<0.001
Median Temperature (°C)	36.9 (36.6-37.2)	36.8 (36.5-37.2)	0.085
Median HR (bpm)	84.0 (72.0-100.0)	72.0 (64.0-88.0)	<0.001

**Figure 3:**
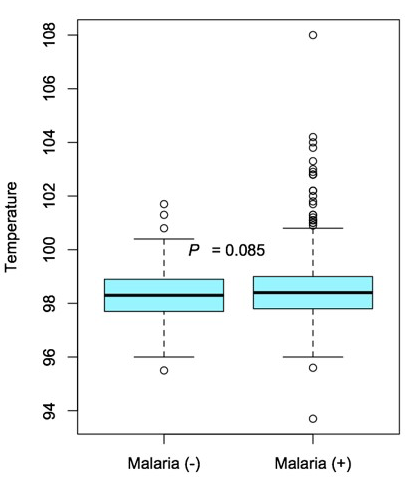
Box and whisker diagramme of the non-significant difference (P=0.085) in median body temperature (bold horizontal line) of the malaria-positive (right) and malaria-negative (left) groups. The interquartile range (box), interquartile range x 1.5 or furthest datum (dotted lines), and any outliers (open circles) are shown.

**Figure 4:**
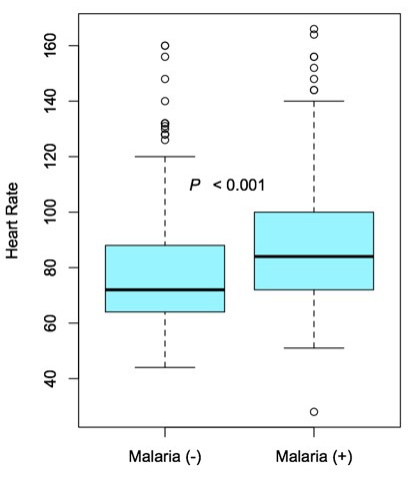
Box and whisker diagramme of the noted significant (P<0.001) difference in median heart rate (bold horizontal line) of the malaria-negative (left) and malaria-positive (right) groups. The interquartile range (box), interquartile range x 1.5 or furthest datum (dotted lines), and any outliers (open circles) are shown.

The median temperature of malaria-positive patients was 36.9°C (36.6-37.2°C) while that of their malaria-negative counterparts was 36.8°C (36.5-37.2°C) (P=0.085) (Figure 3). A more notable difference was observed in heart rate (HR), with a median HR of 84.0 beats per minute (bpm) and 72.0 bpm in the malaria-positive and negative groups, respectively (P<0.001; Table 2, Figure 4). All patients who tested positive by RDT received ACT medication in the pharmacy prior to discharge.

## Discussion

The data obtained provides insight into the prevalence and burden of malaria in the Rorya region of northern Tanzania. According to our results, the prevalence of malaria is 35.8% higher in the Rorya district in comparison to the national average [2]. Many etiologies could factor into this discrepancy including increased *Anopheles* exposure in the district, lack of access to healthcare, patient education, limited disbursement of malaria prevention (ITNs, IPTp, etc.), or outright lack of access to malaria prevention. Regardless, it is evident that individuals within the District are at greater risk of contracting malaria than other Tanzanians. Resource allocation is critical to the success of malaria prevention and treatment. Our data suggests that Rorya, and other regions with high malaria prevalence, warrant an increased allocation of healthcare resources.

It appears that women and children are at greater risk of contracting malaria than their male counterparts. Specifically, children were found to be at a significantly (P<0.001) heightened risk of malaria positivity. The reasons for this noted difference are multi-factorial, with social risk factors and the domestic division of labour playing roles. An analysis by Cotter *et al.* [6] showed that women and children, who are often tasked with water collection early in the morning before sunrise, may suffer from increased exposure to mosquito biting at that time. Others have shown that while women are more likely than men to use ITNs and other preventative measures, they often lack the resources and financial decision-making power in the home to implement these measures [7]. Our analysis noted that there were more women who reported to the two clinic sites in the study. Because the difference in malaria rates between sexes was not significant (P=0.184), the noted difference in this study could be related to sampling error.

A statistical significance was also observed in the percentage of malaria cases between the two clinic sites. Human factors, including patient education and preventive practices, may differ between the Roche and Nyambogo communities. ITN use, antenatal care attendance, medical prophylaxis, and patient education could be quantitatively measured in future site visits to evaluate whether a significant difference exists between the two communities. Factors related to micro-climate and topography may also be involved. An analysis of malaria cases in Indonesia by Hasyim *et al.* [8] yielded that increased rainfall was positively associated with malaria case numbers while altitude and distance from the forest were negatively correlated with malaria prevalence. Interestingly, they also found that distance from a river/major water source had no significant bearing on malaria case rates. While the topographical and precipitation data from the two sites is not readily available, it is plausible that decreased rainfall and/or higher altitude could play a role in the lower malaria case rate noted in Roche.

Analysis of intake vitals uncovered a statistically significant difference in HR between the malaria-positive and malaria-negative groups. Similarly, a study by Anigbogu *et al.* [9] showed predictable changes in HR, rhythm, and blood pressure in malaria patients. While RDT has been shown to be non-inferior to the gold standard of malaria detection [3], their cost is not insignificant and supplies can be limited. In patients with an intermediate pre-test probability of malaria infection, certain HR thresholds could be used as a triaging tool to identify patients that warrant further investigation by RDT.

Tanzania has made impressive progress in malaria-related outcomes over the last fifteen years [2]. Perhaps their largest victory has been in treatment during the peri-natal period, in which Tanzania leads African nations in the percentage of women receiving 3-doses of ACT during pregnancy (>50%) [1]. The associated decrease in vertical transmission rates, increased cost-sustainable preventative measures, and increased access to medical therapy have translated into a decrease in Tanzania’s malaria-associated morbidity and mortality.

## Conclusion

RDT data yields accurate local/ regional prevalence data that can drive more appropriate distribution of resources. As more communities and organizations utilize RDT and register their results with national/international bodies, a more accurate malaria census will be possible.

## References

[1] World Health Organization: (2019). World Malaria Report 2018.. http://https://apps.who.int/iris/handle/10665/330011..

[2] President's Malaria Initiative: Tanzania Country Profile.. http://https://www.pmi.gov/docs/defaultsource/default-document-library/country-pro-files/tanzania_profile.pdf?sfvrsn=18.

[3] Faye B, Nath-Chowdury M, Tine RC, Ndiaye JL (2013). Accuracy of HRP2 RDT (Malaria Antige7n P.f®) Compared to Microscopy and PCR for Malaria Diagnosis in Senegal.. Pathog. *Global Health*.

[4] Wongsrichanalai C, Barcus MJ, Muth S, Sutamihardja A (2007). A review of malaria diagnostic tools: microscopy and rapid diagnostic test (RDT).. Am. J. Trop. Med. Hyg..

[5] Thawer SG, Chacky F, Runge M, Reaves E (2020). Sub-national stratification of malaria risk in mainland Tanzania: a simplified assembly of survey and routine data.. Malar. J..

[6] Cotter C, Sturrock HJ, Hsiang M, Liu J (2013). The changing epidemiology of malaria elimination: New strategies for new challenges.. Lancet.

[7] Lampietti J, Poulos C, Cropper M, Haile M (1999). Gender and preferences for malaria prevention in Tigray, Ethiopia.. Policy Research Report on Gender and Development, Working Paper Series.

[8] Hasyim H, Nursafingi A, Hague U, Montag D (2018). Spatial modelling of malaria cases associated with environmental factors in south Sumatra, Indonesia.. Malar J..

[9] Anigbogu CN, Olubowale OA (2004). Effects of malaria on blood pressure, heart rate, electrocardiogram and cardiovascular response to change in posture.. Nig Qt J of Hosp Med..

